# A method for siRNA-mediated knockdown of target genes in RA-induced neurogenesis using P19 cells

**DOI:** 10.1016/j.mex.2025.103177

**Published:** 2025-01-16

**Authors:** Hossein Khodadadi, Hiroaki Taniguchi

**Affiliations:** a Department of Experimental Embryology, Institute of Genetics and Animal Biotechnology of the Polish Academy of Sciences, Postępu 36, Jastrzebiec 05-552, Poland; b African Genome Center, University Mohammed VI Polytechnic (UM6P), Lot 660, Hay Moulay Rachid, 43150, Ben Guerir, Morocco

**Keywords:** Neurogenesis, Embryoid bodies, siRNA-mediated knockdown, P19 cells, A Method for siRNA-Mediated Knockdown of Target Genes in RA-Induced Neurogenesis Using P19 Cells

## Abstract

This study presents a comprehensive protocol for siRNA-mediated knockdown in the differentiation of P19 cells into neuronal-like cells. Utilizing a retinoic acid (RA)-induced neurogenesis model, P19 cells were cultured under specific conditions that facilitated the formation of embryoid bodies (EBs), which were subsequently differentiated into neuronal-like cells. In this investigation, we specifically targeted the Nfe2l1 gene using siRNA transfection to assess the efficiency and effectiveness of our protocol throughout the neuronal differentiation process. Validation of the differentiation was performed through quantitative reverse transcription PCR (RT-qPCR) analysis, measuring the expression levels of key neuronal markers, including Map2 and Pax6 along with the pluripotency marker Oct4. Additionally, the efficiency of the siRNA-mediated knockdown was confirmed by western blot analysis, which demonstrated significant gene silencing at protein levels. These findings underscore the potential of siRNA technology in elucidating gene function during neuronal differentiation and highlight the critical role of targeted gene silencing in advancing neurogenesis research. Furthermore, this study provides a robust and reliable protocol for gene knockdown in neuronal-like cells derived from P19 cells, thereby facilitating further investigations into the intricate molecular mechanisms that govern neurogenesis, neuronal maturation, and overall brain development.

• Developed a novel protocol for targeted gene knockdown in P19 cells during neuronal differentiation.
• Successful silencing of the Nfe2l1 gene during neuronal differentiation, validated by western blot.
• This study provides a reliable protocol for gene knockdown in neuronal differentiation, aiding functional studies of genes in neurogenesis.

## Graphical abstract

**Figure:**
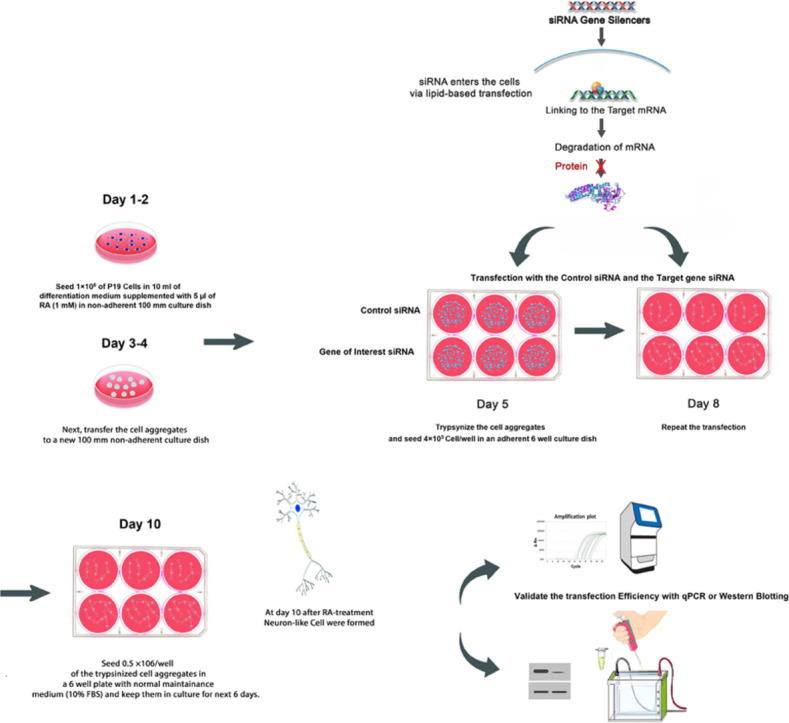

Specifications table

**Table:** 

Subject area:	Biochemistry, Genetics and Molecular Biology
More specific subject area:	Neuroscience
Name of your method:	A Method for siRNA-Mediated Knockdown of Target Genes in RA-Induced Neurogenesis Using P19 Cells
Name and reference of original method:	N*/A*
Resource availability:	N*/A*

## Background

Neuronal differentiation from pluripotent stem cells offers a valuable model for studying neurogenesis and related gene functions. P19 cells, derived from mouse embryonal carcinoma, can be induced to differentiate into neuronal-like cells under the influence of retinoic acid (RA) [[1], [2]]. This process closely mimics early stages of neural development [[3], [4]], making P19 cells a powerful tool for understanding the intricate molecular mechanisms underlying neurogenesis. Such insights are not only critical for advancing our understanding of neural development but also hold significant potential for applications in neurodegenerative disease research. Furthermore, the P19 cell model has been instrumental in elucidating pathways involved in neurogenesis, providing a foundation for therapeutic strategies against neurodegenerative disorders such as Alzheimer's disease and Parkinson's disease [3,[5], [6]].

Small interfering RNAs (siRNAs) are double-stranded RNA molecules that induce the degradation of target mRNA, leading to decreased expression of the corresponding protein [7]. This process, known as RNA interference (RNAi), allows researchers to knockdown specific genes and assess their roles in cellular processes. In the context of neurogenesis, siRNA-mediated knockdown provides a valuable tool to study the impact of individual genes on neuronal differentiation by observing how gene silencing affects cellular behaviour and phenotype [[8], [9]]. Despite the growing use of siRNA technology in various fields of biological research, there is a lack of detailed, standardized protocols for siRNA-mediated knockdown specifically tailored to P19 cells undergoing neuronal differentiation. Many studies have demonstrated the general utility of RNAi for gene silencing [[10], [11]], but the optimization and application of this technique in differentiating neuronal models, such as P19 cells, have not been fully explored. Consequently, there is a pressing need for a well-defined, reproducible protocol that can effectively knock down target genes in these cells and assess the functional outcomes during neurogenesis.

This study aims to establish a reliable protocol for siRNA-mediated knockdown in P19 cell differentiation into neuronal-like cells. By focusing on the knockdown of the Nfe2l1 gene, a transcription factor with important roles in cellular stress responses and neurogenesis, this study demonstrates the efficacy of siRNA in modulating gene expression throughout the neuronal differentiation process. Our goal is to provide researchers with a reliable tool for gene function studies in neurogenesis, enabling further exploration of the molecular mechanisms that govern neural development. Ultimately, this protocol could serve as a foundation for broader applications in neurogenesis research, including the study of neuronal maturation, synaptic formation, and neurodegenerative disease models.

## Method details

### Cell culture

The P19 cell line was cultured in a T25 cell culture flask (surface area 25 cm^2^) with a maintenance medium containing DMEM high glucose with stable glutamine and sodium pyruvate (Biowest, Catalogue number (Cat). #L0130) supplemented with 10 % FBS, 100 units/mL penicillin and 100 units/mL streptomycin) and incubated at 37 °C, 5 % CO2.

For neuronal differentiation, P19 cells were induced with 0.5 µM retinoic acid (RA) (Cat. #R2625; Sigma-Aldrich, St. Louis, MO, USA) in a differentiation medium (same components as the maintenance medium supplemented with 2 % FBS instead of the standard 10 %), for 4 days in a non-adherent culture dish (Cat. #633,180; Greiner bio-one GmbH, Kremsmünster, Austria) to promote the formation of embryoid bodies (EBs). Subsequently, EBs were plated onto adherent culture dishes and allowed to further differentiate in maintenance medium for an additional 6 days [1].

Lipofectamine™ RNAiMAX (Thermo Fisher Scientific, Cat. #13,778,075) and Optimem were used to transfect the siRNA of gene of interest (for our study we used mouse Nfe2l1 siRNA (siNfe2l1), Sense: GCCUGUAGAAGAAUUCAAU, Antisense: AUUGAAUUCUUCUACAGGC), and Negative Control siRNA (siNC) (MISSION® siRNA Universal Negative Control, Sigma-Aldrich, Cat. #SIC-001) to the cells.

#### Generation of embryoid bodies (Day 0–4)

• Prepare 10 mL of differentiation medium with 5 µL of 1 mM RA (final concentration: 0.5 µM RA).
• Seed 1 × 10^6^ of P19 cells in 100 mm non-adherent dishes.
• Incubate at 37 °C, 5 % CO2 for 2 days.
• Change the differentiation medium with a fresh one after 2 days and, induce the RA again and transfer the EBs to a new non-adherent dish for an additional 2 days of incubation under the same conditions.

#### Formation of neuronal-like cells (Day 5–10)

• On Day 5, transfer the EBs to a 15 mL tube, allow them to settle for 3 min, aspirate supernatant, and wash with serum and antibiotic-free DMEM.
• After the EBs are settled, aspirate supernatant, add 2 mL trypsin-EDTA (0.25 %), and incubate in a 37 °C water bath for 12 min.
• Next, add 4 mL maintenance medium to stop trypsinization and centrifuge at 1000 x g for 2 min.
• Suspend the cells in 5 mL of maintenance medium and determine the cell number with an automated counter.
• Perform siRNA-mediated knockdown (as explained in siRNA-mediated knockdown section)
• Seeding: 0.4 × 10^6^ cells/well in 6-well plates with 2 mL maintenance medium.
• Incubation: 37 °C, 5 % CO2 until day 8.
• Repeat the siRNA transfection on adherent cells (as explained in siRNA-mediated knockdown section).
• Incubation: 37 °C, 5 % CO2 until day 10.

### *siRNA-mediated knockdown*

• Prepare transfection mixtures as follows to achieve a final concentration of 20 nM of siRNA, based on the manufacturer's instructions:

Opti-MEM, RNAiMAX, and Target siRNA (20 nM final concentration)

Opti-MEM, RNAiMAX, and Control siRNA (20 nM final concentration)

• Vortex and spin down, incubate at room temperature for 15 min, and then take all of the mixture and add to its appropriate well in a 6-well plate,
• Next, add 0.4 × 10^6^/ml of cells from the last section to the mixture and top up the volume to 2 ml with maintenance medium.
• Incubate for 48 h, 37 °C, 5 % CO2 (Optional: you can replace the medium with normal medium containing P/S after 24 h incubation)
• At day 8 of differentiation, aspirate the medium and wash the cells with pre-warmed 1xPBS gently
• Repeat the transfection procedure for the adherent cells, as previously described. Ensure that the transfection mixture is added carefully to avoid disrupting the adherent cells.
• Important note: While adding any reagent, including the PBS, medium, and transfection mixture, please be careful not to disturb the adherent cells; we are recommending to point the wall of each well with the pipet and release the reagents very gently.
• Extract Protein or RNA to check the efficiency of your knockdown.
• Confirm the transfection efficiency and knockdown levels by western blotting or RT-qPCR.

### Microscopic observation

In order to track morphological changes, we observed and pictured the formation of cell aggregates and neuron-like cells, respectively, at days 4 and 10 of RA-induced neurogenesis via an inverted microscope (Nikon Eclipse TE2000-S) equipped with Nomarski differential interference contrast (DIC).

### Isolation of RNA and RT-qPCR

• On day 10 of neurogenesis, using the NucleoSpin RNA Mini kit for RNA purification (MACHEREY-NAGEL, Cat. #740,955.50), the total RNA was isolated from all the Neuronal-like cells.
• Next, we used 0.5 µg of total RNA to produce cDNA by the NG dART RT kit according to the manufacturer's instructions (EURx, Cat. #E0801).
• RT-qPCR was performed to measure the expression levels of Map2 (neuronal marker), Pax6 (neuronal progenitor marker), Oct4 (pluripotency marker), and β-Actin (control gene). The reactions utilized SYBR Green Master Mix (Cat. #2017–100HS, A&A Biotechnology) on the Roche LightCycler^Ⓡ^ 480 System. The cycling conditions were as follows: an initial incubation at 95 °C for 5 min, followed by 40 cycles of 95 °C for 10 s, 60 °C for 10 s, and 72 °C for 10 s. Relative expression levels of target genes were normalized to β-Actin and analyzed using the 2^(-ΔΔCt) method. Primer sequences for the target genes were designed and validated for specificity and efficiency, as listed in Table 1.

**Table 1:** Primers used for RT-qPCR.

Gene of Interest	Primer Sequence	Product Size (bp)
*mMap2*	F: GCTGAGATCATCACACAGTC R: TCCTGCCAAGAGCTCATGCC	211
*mOct4*	F: GGCGTTCTCTTTGGAAAGGTGTTC R: CTCGAACCACATCCTTCTCT	313
*mPax6*	F: TAGCCCAGTATAAACGGGAGTG R: CCAGGTTGCGAAGAACTCTG	132
*mβ-Actin*	F: GTACCACCATGTACCAGGC R: AACGAGTCGATAACAGTC	247

### Western blotting

• Protein samples were extracted using 250 µl of T-PER buffer/well (Cat. #78,510, Thermo Fisher Scientific, Waltham, MA, USA). The concentration of proteins was determined using a BCA assay (Cat. #23,225, Thermo Fisher Scientific, Waltham, MA, USA).
• Equal amounts of protein (20 µg) were then separated by SDS-PAGE and transferred to PVDF membranes (Cat. #03,010,040,001, Sigma-Aldrich, Saint Louis, MO, USA).
• The membranes were blocked with 5 % (w/v) non-fat dry milk to prevent non-specific binding in RT for 15 min. Following blocking, the membranes were probed with primary antibodies against NFE2L1 (Cat. #8052, Cell Signaling Technology, Danvers, MA, USA, 1:1000) and β-Actin (Cat. #4970, Cell Signaling Technology, Danvers, MA, USA, 1:1000) as a loading control, and incubated in a shaking incubator at 4 °C overnight.
• After primary antibody incubation, membranes were incubated with HRP-conjugated secondary anti-rabbit IgG antibody for NFE2L1 and β-Actin (Cat. #A6154, Sigma-Aldrich, Saint Louis, MO, USA, diluted 1:5000).
• Finally, The signals were detected using an ECL (Cat. #RPN2235, GE Healthcare, Amersham Place, UK) substrate and visualized with Bio-RAD ChemiDoc MP chemiluminescence imaging system.

## Method validation

### Morphological validation of neurogenesis

**Fig. 1:**
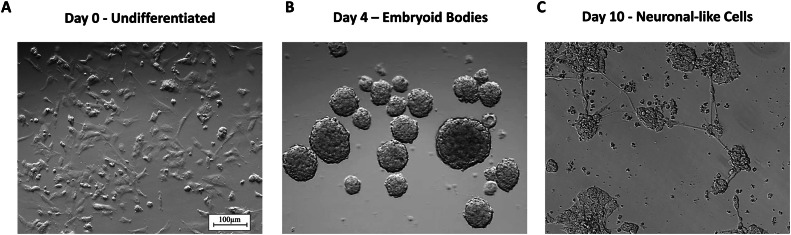
**Representative images of analysis of P19 cell line RA-induced neurogenesis.** (A) Light microscopic image of undifferentiated P19 cell line. (B) Light microscopic image of embryoid bodies formation at day 4 and (C) formation of neuronal-like cells at day 10 of neurogenesis following initial RA-treatment (0.5µM) and embryoid bodies formation stage. Scale bar =100 µm.

Morphological validation was also carried out by observing and imaging the formation of EBs and neuronal-like cells at days 4 and 10 of RA-induced neurogenesis compared to undifferentiated P19 cells (Fig. 1. A-C). This was performed using an inverted microscope (Nikon Eclipse TE2000-S) with Nomarski differential interference contrast (DIC). These images provided visual confirmation of the development of neuronal-like cells and the overall progress of differentiation.

### Molecular validation of neurogenesis

**Fig. 2:**
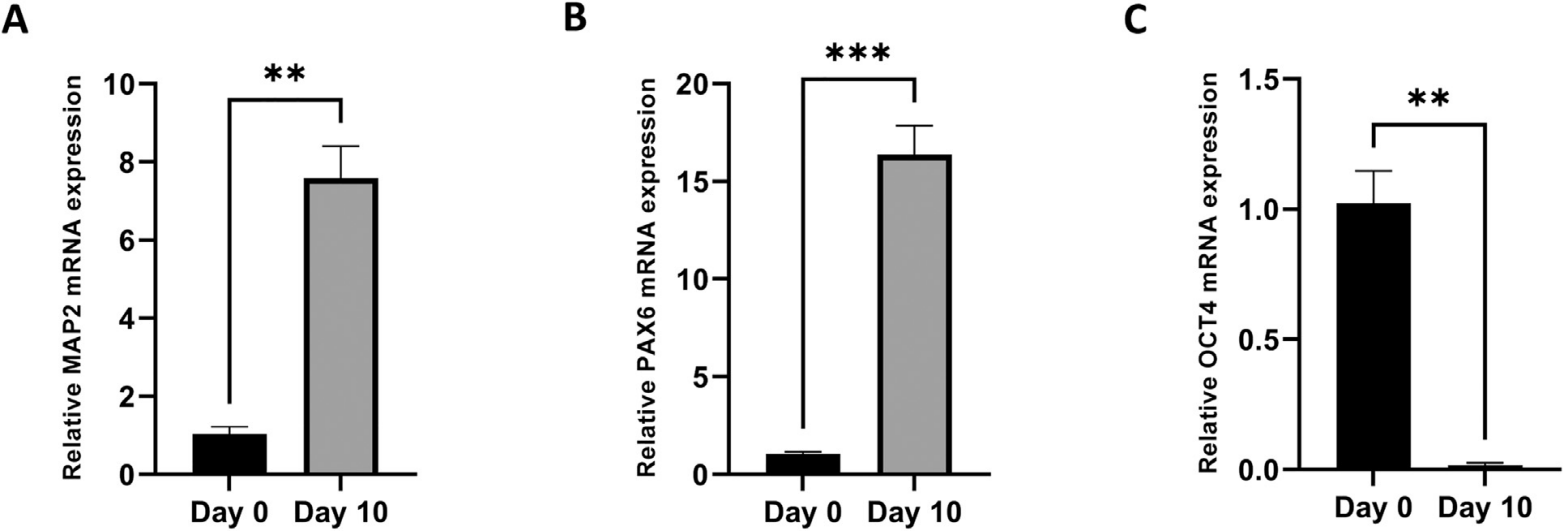
**Changes in Gene Expression in the P19 Cell Line During Neurogenesis.** The bar graph shows gene expression in undifferentiated P19 cells (Day 0, control) compared to day 10 of RA-induced neurogenesis. The mRNA levels of neuronal markers (A) Map2 and (B) Pax6, as well as the pluripotency marker (C) Oct4, were evaluated using RT-qPCR, with β-Actin serving as the reference gene. Statistical significance are indicated by **p < 0,01 andand *** *p* < 0.001. Error bars represent the mean ± *S*.E.M. (*n* = 3 per group). A T-test was used for statistical analysis.

To validate our differentiation protocol, we assessed the expression of key markers associated with neurogenesis and pluripotency. Using RT-qPCR, we analyzed the levels of Map2 (Fig. 2. A), Pax6 (Fig. 2. B), and Oct4 (Fig. 2. C) in both differentiated and undifferentiated P19 cells.

Our results demonstrated a significant increase in the expression levels of Map2 and Pax6 in differentiated cells compared to undifferentiated controls, indicating successful neuronal differentiation. Conversely, the expression of Oct4 significantly decreased in differentiated cells, further confirming the loss of pluripotency and the progression toward a neuronal lineage.

### siRNA-mediated knockdown validation

Having confirmed that neurogenesis was effectively induced, we proceeded to test our siRNA-mediated knockdown method. Cells were transfected at two-time points, days 5 and 8, of neuronal differentiation. To validate the efficiency of siRNA-mediated knockdown of NFE2L1 in differentiated neuronal cells, we performed western blot analysis. NFE2L1 protein levels were assessed in both negative control and NFE2L1 siRNA-transfected cells. Given that NFE2L1 protein levels are augmented upon proteasome inhibition, we treated the cells with Mg132 (25 µM) for 2 h before protein extraction on day 10 of differentiation.

**Fig. 3:**
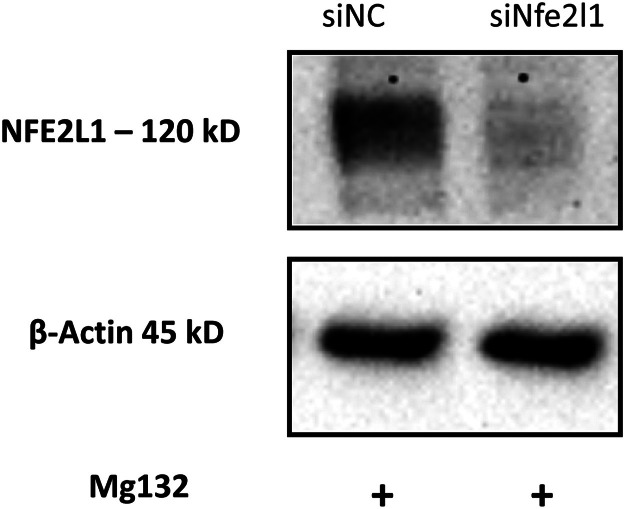
**Validation of siRNA-mediated knockdown of NFE2L1 in differentiated neuronal cells.** Western blot analysis showing the efficiency of NFE2L1 knockdown in neuronal cells transfected with NFE2L1-specific siRNA (siNfe2l1) and Negative siRNA control (siNC). Cells were treated with Mg132 (25 µM) for 2 h before protein extraction on day 10 to enhance the detection of NFE2L1.

The Western blot results revealed dramatic reduction in NFE2L1 protein levels (Fig. 3) in the siRNA-transfected neuronal cells compared to the control, confirming the efficacy of the siRNA-mediated knockdown. These findings demonstrate that our protocol effectively induces neuronal differentiation and allows for targeted gene knockdown, providing a reliable model for studying gene function in neurogenesis.

### Limitations

**Cell Line Specificity:** The protocol is optimized for P19 cells and may not directly translate to other cell lines without significant adjustments, particularly in neuronal differentiation efficiency.

**siRNA Efficiency:** The effectiveness of siRNA-mediated knockdown can vary based on the transfection reagent used, the siRNA sequence, and the gene target. Optimization may be needed for different genes or experimental conditions.

## Ethics statements

This is an in vitro study, and confirmation of its compliance with the ethical standards for experimental works on animals is not necessary.

## CRediT authorship contribution statement

**Hossein Khodadadi:** Methodology, Validation, Formal analysis, Visualization, Data curation, Writing – original draft. **Hiroaki Taniguchi:** Conceptualization, Supervision, Project administration, Funding acquisition, Writing – review & editing.

## Declaration of competing interest

The authors declare that they have no known competing financial interests or personal relationships that could have appeared to influence the work reported in this paper.

## Data Availability

Data will be made available on request.
